# Introduction of Carbonyl Groups into Antibodies

**DOI:** 10.3390/molecules28237890

**Published:** 2023-12-01

**Authors:** Evgeny L. Gulyak, Vera A. Alferova, Vladimir A. Korshun, Ksenia A. Sapozhnikova

**Affiliations:** Shemyakin-Ovchinnikov Institute of Bioorganic Chemistry, Miklukho-Maklaya 16/10, 117997 Moscow, Russia; evgeny.gulyak@gmail.com (E.L.G.); alferovava@gmail.com (V.A.A.); v-korshun@yandex.ru (V.A.K.)

**Keywords:** antibodies, carbonyl groups, oxime ligation, UAAs, periodate oxidation, ADC, glycans, biorthogonal reactions, glycans oxidation, immunoglobulins modification

## Abstract

Antibodies and their derivatives (scFv, Fabs, etc.) represent a unique class of biomolecules that combine selectivity with the ability to target drug delivery. Currently, one of the most promising endeavors in this field is the development of molecular diagnostic tools and antibody-based therapeutic agents, including antibody–drug conjugates (ADCs). To meet this challenge, it is imperative to advance methods for modifying antibodies. A particularly promising strategy involves the introduction of carbonyl groups into the antibody that are amenable to further modification by biorthogonal reactions, namely aliphatic, aromatic, and α-oxo aldehydes, as well as aliphatic and aryl–alkyl ketones. In this review, we summarize the preparation methods and applications of site-specific antibody conjugates that are synthesized using this approach.

## 1. Introduction

Conjugates of biomolecules [1,2] serve as potent research tools in molecular biology and find applications as therapeutic agents [3]. A variety of reactions from the arsenal of organic chemistry are used to obtain them [1,2,4,5]. The modification of biomolecules should be specific to particular functional groups [2]. The key conjugate assembly reaction should be conducted with high yield in aqueous solutions at physiological temperatures and should not affect other functional groups present in the biomolecule [6,7]. Requirements for such reactions were established at the beginning of the century, leading to the pursuit of new “bioorthogonal” reactions [8] and enhancements of existing methods. The 2022 Nobel Prize in Chemistry was awarded for the development of these reactions, a testament to their significant practical importance [9,10,11,12].

**Figure 1:**
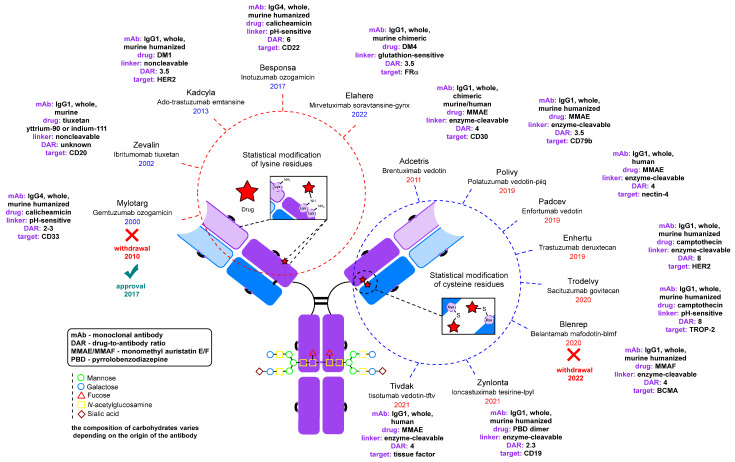
FDA-approved antibody–drug conjugates and radioimmunoconjugates [24,25,26,27,28,29,30,31,32,33,34,35,36,37,38,39,40,41,42,43,44,45,46,47,48,49,50,51,52,53,54,55,56,57,58,59,60,61,62,63,64,65,66,67,68,69,70,71,72].

Antibodies [13,14] and their derivatives [14,15,16,17] have long been used for diagnostics [18,19,20] and therapy [21,22]. The chemical modification of antibodies has significantly broadened their scope of application [23]. The primary focus of antibody chemical modification is in the development of therapeutic antibody–drug conjugates (ADCs) (Figure 1) [24].

ADCs are a rapidly growing class of drugs for the treatment of oncologic diseases [73,74]. Every year the U.S. Food and Drug Administration (FDA) approves new conjugates for clinical use [75].

There are five classes of mammalian immunoglobulins (Ig): IgA, IgE, IgM, IgG, and IgD [76]. They differ from each other in structure, function, and localization in the body. While some attempts have been made to use IgM [77,78] and other types in conjugation [78] and therapy [79], the IgG class has been by far the most popular for therapeutic purposes as a monoclonal antibody, and, in particular, for the construction of ADCs. The IgG class is the most abundant in mammals and has high stability and a long circulation time in the bloodstream [13]. In this work, we will focus on conjugates involving immunoglobulins of this isotype and their derivatives, such as nanoantibodies.

The IgG class is divided into four subtypes: IgG1, IgG2, IgG3, and IgG4. Of these, the IgG1 subtype remains the most popular subtype for ADC development due to its excellent stability (~21 days circulation time in the bloodstream) and its ability to recruit the complement system for enhanced therapeutic efficacy. IgG2 is less effective in triggering the complement system and is less stable, as this subtype can form covalent dimers leading to subsequent aggregation. IgG3, on the other hand, is less suitable for therapeutic use due to its short plasma half-life (~7 days). IgG4 can form hybrid, bispecific antibodies with non-identical paratopes in their two Fab (fragment antigen-binding) regions by exchanging one pair of light and heavy chains with other IgG4 antibodies [80,81], which hinders the targeting ability of the antibodies of this subtype.

Engineered bispecific antibodies are promising for therapeutic applications as well as for ADC development [82,83]. Bispecific antibodies are the subject of numerous literature reviews [84,85,86,87,88]; however, we will not focus on them here as almost all of them are modified with a genetically introduced [89] or native cysteine residue [90] using maleimide reagents [91], which is outside the scope of this review.

To overcome problems related to the immunogenicity of antibodies [92,93] derived from organisms of different species, recombinant antibodies—chimeric and humanized [94]—are widely used [80,93]. It is worth noting that the majority of antibodies used in FDA-approved ADCs [24] are humanized murine antibodies [13,25].

A fundamental property of antibodies is their ability to recognize and bind to specific antigens [76]. It is important to emphasize that any modification introduced to an IgG molecule should be carefully designed and characterized to prevent any adverse impact on the antibody’s antigen binding ability, specifically on the antigen-binding site [14]. This is particularly important for ADCs, as the precise recognition of the tumor antigen is essential for the targeted delivery of cytotoxic agents [95].

**Figure 2:**
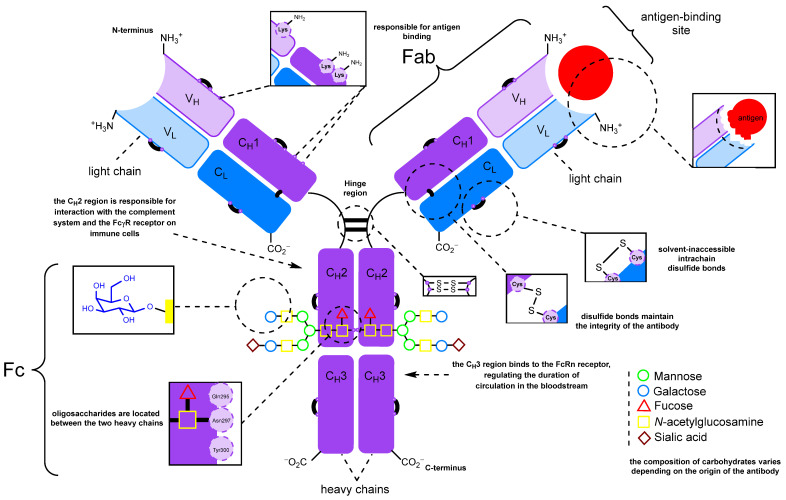
The structure of immunoglobulin G and the sites available for modification.

The schematic structure of a typical full-length monoclonal antibody of the IgG class is shown in Figure 2. Immunoglobulin G is a Y-shaped protein molecule with a size of about 10 nm and a mass of about 150 kDa. It is composed of four polypeptide chains. The two larger chains, each consisting of two domains of roughly 110 amino acids, are referred to as heavy chains. These heavy chains are connected by a flexible polypeptide chain rich in cysteine and proline known as the hinge region [76,96]. Within the hinge region, the two heavy chains are linked to each other by disulfide bonds (ranging from 2 to 11, depending on the IgG subtype) that are exposed to the surrounding solvent [97]. In addition, two light chains, each consisting of a single domain, are linked to one domain of a heavy chain by disulfide bonds (one bond between one heavy and one light chain in all IgG subtypes) [97]. This link forms Fab fragments, which are directly responsible for binding to antigens. It is important to note that this structural arrangement provides the antibody molecule with exceptional flexibility. Preserving this structure during the modification process is crucial. The Fab regions can rotate about their axes, move in different directions at significant angles, bend, stretch, and contract to effectively engage with antigens [96,98]. Thus, the angle between the Fab regions modulates the accessibility of the antigen, and perturbations introduced during modification can reduce affinity. The same considerations apply to the Fc (fragment crystallizable) region, but in the context of its effector properties, such as interactions with the immune system. These properties may be less important when obtaining fluorescently labeled or therapeutic antibody–drug conjugates, where the sole function of the antibody is targeted delivery.

Figure 2 also shows the functional groups present in native antibodies that are available for modification. These include N-glycans, side chains of lysine and other amino acids, and disulfide bonds. It is important to note that disulfide bonds can be further categorized into interchain bonds located in the hinge region, which are relatively accessible for modification, and intrachain bonds, of which there are 12 in all IgG subtypes, which are positioned on the surface of the antibody molecule [97,99]. The first FDA-approved ADC, Mylotarg, was synthesized by statistical modification of the lysine side chains. Subsequently, approaches to controlled modification using thiol groups generated by partial chemical reduction [100] of interchain disulfide bonds were developed (Figure 2) [101]. Cysteine labeling has become a widely adopted strategy, with numerous reviews devoted to its characteristics [99,102,103,104]. Notably, among the ADCs that have been reported or are currently in clinical trials, various antibody modification approaches have been explored, including those leveraging genetic engineering techniques [75].

Among the various bioorthogonal reactions available, those involving carbonyl groups are particularly intriguing for several reasons [105]. Carbonyl groups (aldehydes and ketones) (1) are not found in major classes of biopolymers such as proteins, nucleic acids, and polysaccharides; (2) can be site-specifically introduced into biomolecules; and (3) are well suited for bioorthogonal modification reactions, including oxime ligation [106], hydrazone formation [106] or the Pictet–Spengler reaction [105]. Carbonyl groups (aliphatic, aromatic, and α-oxo aldehydes, as well as aliphatic and aryl-alkyl ketones) can be incorporated into immunoglobulin molecules using various methods. This review will focus on the preparation and application of site-specific antibody conjugates, whose synthesis involves the introduction of carbonyl groups into the antibody molecule and their subsequent modification by biorthogonal reactions.

## 2. Methods for Introducing Carbonyl Groups into Antibodies

**Figure 3:**
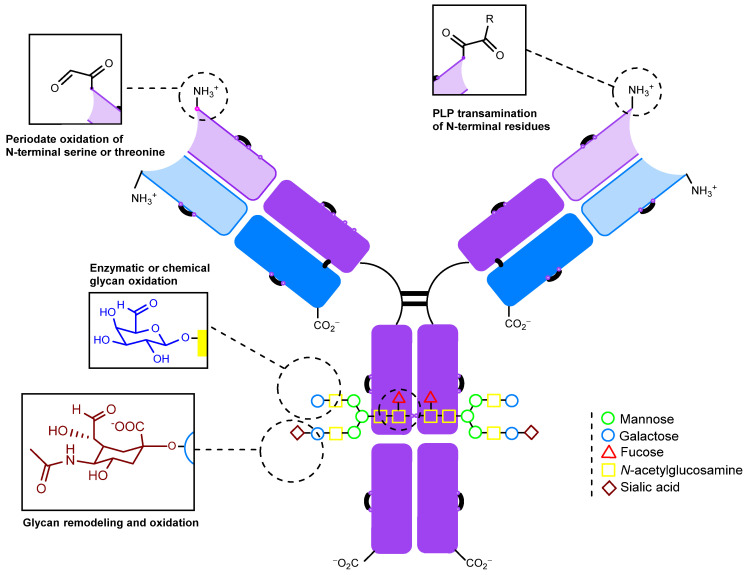
Sites for modifications that do not require genetic engineering.

There are currently a large number of methods for introducing a carbonyl group into an antibody molecule. Some of these methods have been known for a long time (e.g., periodate oxidation [77,78,107]) and were originally developed for other applications [108], but were later adapted for immunoglobulins and other glycosylated proteins [78]. Some approaches are relatively new (e.g., genetically engineered amino acid introduction [109]) and somewhat complicated. Many of the approaches can be used in conjunction with each other (e.g., glycoengineering methods combined with periodate oxidation), so their division into groups may be tentative. Nonetheless, we have chosen to categorize the approaches into two groups: firstly, the more general approaches that only exploit the natural modification capabilities of antibodies (Figure 3) and secondly, approaches that rely on genetic engineering to alter the structure of the antibody.

### 2.1. Introduction of the Carbonyl Group into Native Antibodies

#### 2.1.1. Periodate Oxidation of Glycans

IgG molecules typically contain 2–3% carbohydrates by mass. These carbohydrates are present as a branched oligosaccharide chain, conservatively linked to the asparagine-297 residue (Asn297, N297, Figure 2) in the C_H_2 region of the Fc heavy chain of the immunoglobulin fragment [110]. The localization of carbohydrates on a serine or threonine residue of IgG is rare [111]. As a result, oligosaccharides are consistently located at a significant distance from the antigen-binding center [112]. Numerous reviews have been devoted to the properties and functions of immunoglobulin glycans and their structure [110,111,113,114,115,116,117,118,119,120,121,122,123,124,125]. Hence, the oxidation of oligosaccharide fragments of antibodies to form carbonyl groups appears to be a promising method for initial modification, which can then be followed by the conjugation of the desired molecule [112,126].

**Figure 4:**
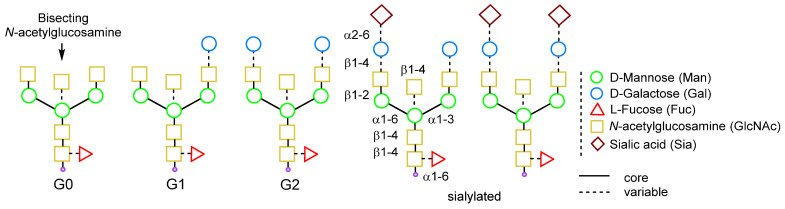
Human IgG glycoforms [110].

The carbohydrate composition is usually limited to fucose, mannose, galactose, *N*-acetylglucosamine, and sialic acid (Figure 4). The oligosaccharide structures and possible isoforms depend on the antibody producer and can be predicted [127,128]. For example, CHO cells produce antibodies with varying profiles of glycosylation [129,130,131]. However, obtaining antibodies with a homogeneous isoform of glycans in cellular expression systems is challenging. This is due to the various cellular processes, including the activity of enzymes such as glycosidases, glycosyltransferases, and others [111].

For the oxidation of glycans, the reaction with sodium metaperiodate (NaIO_4_) is suitable [132]. This reaction has been known since 1928 and was originally developed by Léon Malaprade for the selective oxidation of vicinal alcohols [133] and β-amino alcohols [134].

In the case of glycans, the vicinal diols of sugars undergo oxidation, and *cis*-diols are oxidized much more readily than *trans*-diols. Thus, of the sugar residues listed above, only sialic acid, galactose, fucose, and, to a lesser extent, mannose residues can be oxidized. In some reports, mannose did not undergo oxidation under the most favorable conditions (10–20 mM) [135].

**Figure 5:**
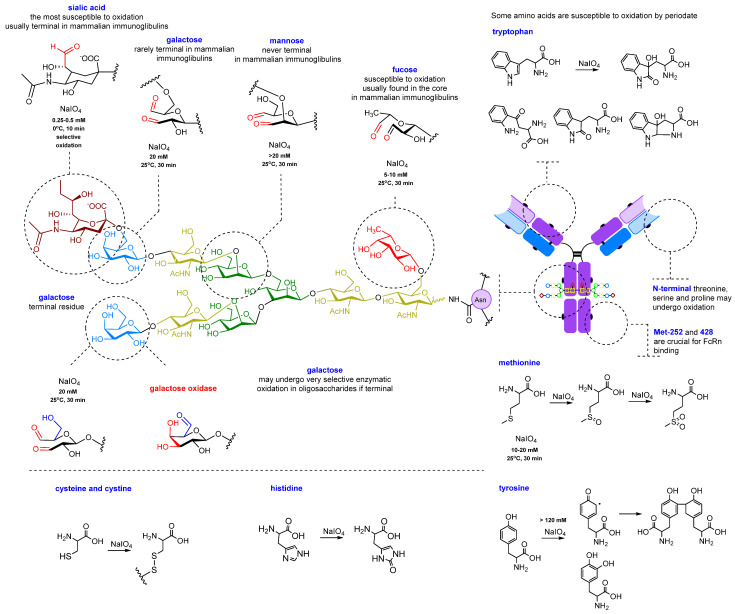
Glycan oxidation and possible side reactions.

Periodate oxidation is influenced by many factors, including the concentration of the oxidant, pH, temperature, and reaction time [136]. Under mild conditions, oxidation may selectively target sialic acid residues, resulting in the generation of an aldehyde group at its exocyclic C-7 atom. Under more forcing conditions, it may affect other carbohydrate residues with vicinal diols, or undesirably oxidize sensitive amino acids such as tryptophan, tyrosine, and methionine [107] (Figure 5).

**Figure 6:**
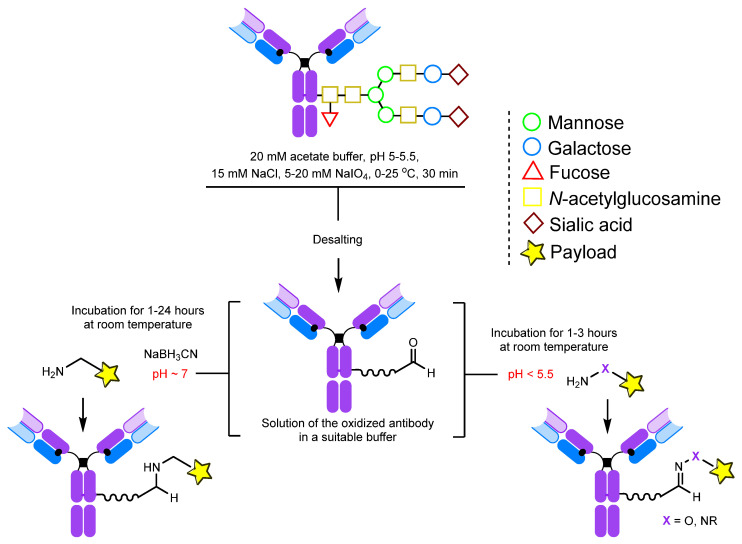
Conditions for periodate oxidation of glycans and further modification [139,155].

The optimal conditions for selectively oxidizing oligosaccharides alone involve running an oxidation for 30 min with a NaIO_4_ concentration of 5–20 mM at a temperature of 25 °C in an acetate buffer with a pH of 5–5.5 [135,137,138] (Figure 6). These conditions can be adjusted to achieve a specific level of carbonyl group introduction via the oxidation of other sugar moieties [136,139,140]. Theoretically, a maximum of 24 to 28 aldehyde groups can be generated per antibody through glycan oxidation (Figure 5). However, due to steric factors and side reactions, not all of these groups can be further modified; for instance, hydrazides form cyclic adducts with two neighboring aldehyde groups instead of two individual hydrazones, which reduces the degree of modification. As a result, the degree of labeling usually varies from 2 to 10 molecules per antibody [139].

At high concentrations of periodate (>50 mM), dimers can readily form and there is a possibility of oligomer formation, especially at higher temperatures and more alkaline pH values. This is primarily due to the reaction of aldehyde groups with ε-amino groups of lysine, resulting in the formation of Schiff bases which is favored under basic conditions. In addition, the polymerization of antibodies via the Schiff bases formation can be observed [136,139,141]. If amino acids in the antigen-binding site are involved in dimer formation, this can lead to a significant loss of affinity [139]. Additionally, at concentrations of sodium periodate above 100 mM, a process called overoxidation can occur, in which oligosaccharides are degraded and detached from the antibody [107]. Since one of the functions of oligosaccharides is to increase the solubility of immunoglobulins, antibodies may aggregate after the loss of oligosaccharide chains [139,142,143].

At periodate concentrations above 50 mM, sensitive amino acids also undergo destructive processes, resulting in decreased affinity and an overall loss of immunoglobulin stability [107,135,136,142,144,145,146,147,148,149,150,151,152,153]. It should be noted that some antibodies are more sensitive to oxidation and lose their ability to bind very easily even at low concentrations of sodium periodate (5–10 mM) [136]. This is due to the location of oxidation-sensitive amino acids in the antigen-binding site. For such antibodies, even milder oxidation conditions should be selected, such as less than 0.5 mM sodium periodate, cooling to 0 °C and shorter incubation times [136]. Prolonged incubation can almost completely abolish the affinity [142].

It is important to note that selective oxidation of sialic acid residues with periodate uses much lower concentrations of periodate (0.25–0.5 mM) than conventional oxidation of oligosaccharides [107,136]. These conditions are the most suitable for sensitive amino acids. Oxidation of methionine residues [143,150], including residues Met-252 and Met-428 located on the Fc region of C_H_3 close to the binding site of the neonatal receptor FcRn (the neonatal fragment crystallizable (Fc) receptor) [154], can affect the efficiency of binding to the latter [143]. This receptor is responsible for the recirculation of the antibody from lysosomes back to the intercellular space, as in the case of nonspecific pinocytosis, and a decrease in the interaction with it significantly reduces the lifetime of the immunoglobulin in the blood [154].

This process occurs in the following way: after nonspecific capture by the cell, the antibody enters the acidic lysosome, where it binds to the neonatal receptor at pH below 6. Subsequently, it is released intact into the intercellular space. There, when the pH is raised to neutral values of about 7.4, the antibody is released from the receptor due to the lack of binding. Importantly, in the process, the antibody is protected from enzymatic degradation in the lysosome by binding to FcRn [154].

Partial oxidation (~40%) of the Met-252 residue has little effect on FcRn, causing a 25% decrease in binding. However, a 60% reduction in binding of the antibody to FcRn results in an 80% reduction in immunoglobulin circulation time. It should be noted that the risk of methionine oxidation increases at acidic pH values. At neutral pH values, the oxidation of Met, Tyr, and Trp is almost nonexistent. Thus, performing the oxidation at a pH of 5.0–5.5 represents a compromise between avoiding the undesired amino acid oxidation and preventing the Schiff base formation [107,137].

Temperature also greatly affects the oxidation process, but temperature conditions should be selected individually for each antibody. Most sources mention either oxidation at room temperature or cooling to 0–4 °C [136,137,139,142,156].

It should be noted that for some applications, such as affinity chromatography, periodate oxidation is an excellent solution. This is because the immobilization of the antibody on solid support using oxidized Fc fragment oligosaccharides occurs with the correct orientation of the antibody, making the antigen-binding sites accessible to the target. In the case of immobilization using lysine or cysteine residues, however, the orientation of the antibodies is statistical, rendering some antigen-binding sites inaccessible and leading to a reduction in the overall binding efficiency [156,157,158,159].

Despite the fact that glycan modification affects the effector properties of antibodies, in in vivo tests in mice, ^125^I-tyrosine-labeled radioactive immunoglobulins demonstrated greater efficiency and affinity compared to conjugates that were statistically labeled using lysine or tyrosine residues. While the latter underwent intensive dehalogenation by liver enzymes and subsequent excretion, the glycan-labeled conjugates remained more than 80% intact. This effect is thought to be due to the localization of the glycans in the recess between the two heavy chains of the antibodies, making them inaccessible to enzymes. Similar positive results were obtained for ^111^In-labeled conjugates [112].

#### 2.1.2. Transamination

**Figure 7:**
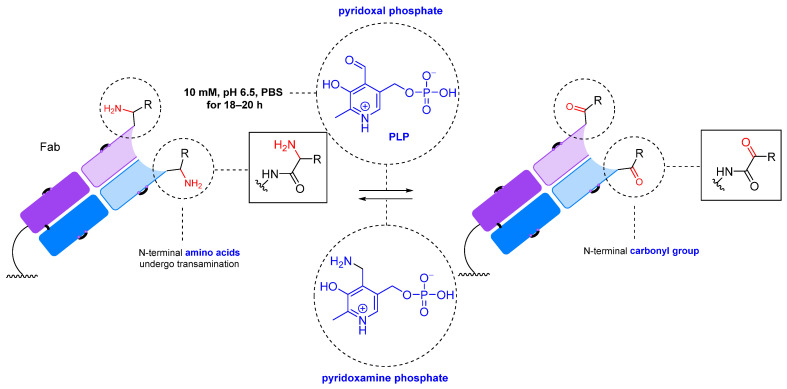
PLP-mediated N-terminal transamination [160].

**Figure 8:**
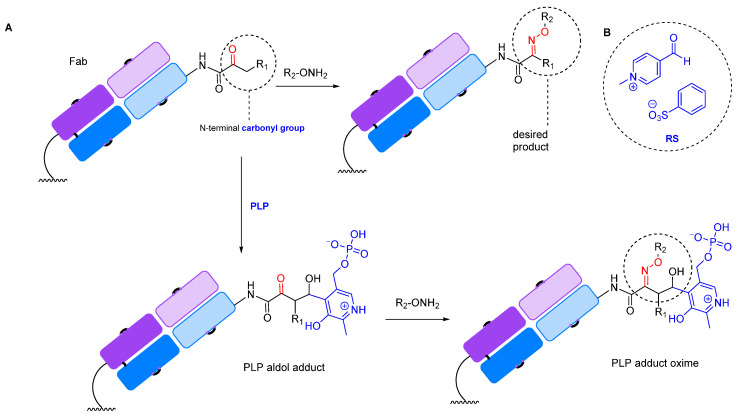
(**A**) Some amino acids form adducts with pyridoxal phosphate [162]. (**B**) N-methylpyridinium-4-carboxaldehyde benzenesulfonate salt (Rapoport’s salt, RS) [163].

Transamination reactions can be used to introduce a carbonyl group at the N-terminus of an antibody of any origin. The reaction involves pyridoxal-5-phosphate (PLP) at 37–50 °C in an aqueous solution (Figure 7) [160,161]. The advantage of this method is its selectivity: it introduces the carbonyl group at the N-terminus without affecting the antigen-binding site. However, a significant disadvantage is that different N-terminal amino acid residues have varying reactivity in the oxidation by pyridoxal-5-phosphate and subsequent modification [162]. For example, alanine, glycine, aspartic acid, glutamic acid, and asparagine form carbonyl products that are easily functionalized by oxime ligation, while the most common N-terminal amino acid, glutamine, is oxidized to a keto derivative that is unreactive toward oxyamines. In addition, some amino acids form adducts with pyridoxal (Figure 8A) [162]. Other disadvantages of this method are the limited number of modification sites (one site per chain, four sites for the whole antibody) and, consequently, a low payload-carrying capacity [160]. In addition, the transamination reaction works best at a temperature of 50 °C, which is undesirable when working with antibodies. Although these limitations hinder the widespread application of this method, it has been successfully applied to create therapeutic drug-loaded Fc antibodies [161].

These difficulties can be overcome by genetic engineering methods, such as incorporating the desired amino acids at the N-terminus of the antibody. Additionally, there have been attempts to create analogs of pyridoxal phosphate that can effectively oxidize the amino acid residues that are unreactive toward pyridoxal. For the selective oxidation of antibodies with genetically engineered glutamine residues introduced at the N-terminus, a PLP analog with better efficacy has been developed: N-methylpyridinium-4-carboxaldehyde benzenesulfonate salt (Rapoport’s salt, RS, Figure 8B) [163].

#### 2.1.3. Glycan Remodeling

This group of methods is based on the introduction of a carbonyl group into an antibody by enzymatic modification of the composition and structure of glycans without additional genetic engineering.

**Figure 9:**
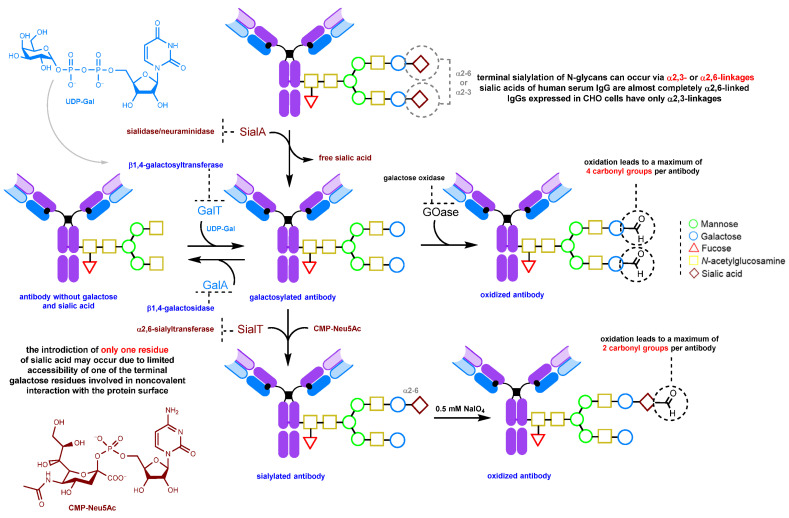
Enzymatic glycan remodeling [107,125,143,152,164,165,166,167].

**Figure 10:**
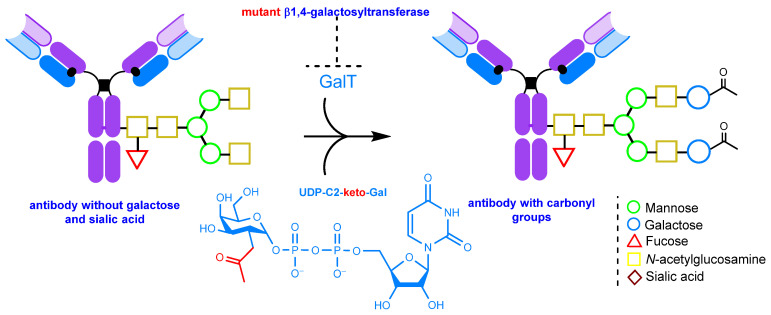
The introduction of C2-keto-galactose into the glycan chain [152,169].

One such approach to glycan modification relies on the use of galactose oxidase (Figure 9 and Figure 10). The enzyme forms an aldehyde group at the C6 position of the terminal galactose or *N*-acetylgalactosamine. However, the method has a significant drawback: in mammalian glycoproteins, the terminal residue is often sialic acid, so additional treatment with neuraminidase is required to make the galactose residues available for modification [107].

Alternatively, terminal sialic acid residues can be enzymatically introduced into the glycan, followed by their mild oxidation using sodium periodate (0.25–0.5 mM) to form carbonyl groups (Figure 9) [143,152]. For trastuzumab, at NaIO_4_ concentrations below 1 mM, there is no significant loss of binding (less than 15%) to the FcRn receptor due to methionine oxidation. However, at concentrations above 4 mM, a 40% loss of binding occurs due to the oxidation of methionine residues, in particular Met-252 [143].

This method is attractive due to very mild oxidation conditions, requiring less than 0.5 mM of sodium periodate for selective sialic acid oxidation. Under these conditions, there is virtually no undesirable oxidation of amino acids, especially methionine, the integrity of which is important for the long circulation time of the antibody. Antibodies oxidized under mild conditions retain their stability and are not prone to aggregation. For trastuzumab, aggregation was observed under harsher conditions (7.5 mM NaIO_4_) [143]. If the Fc glycan lacks terminal sialic acid residues and is galactose- and fucose-poor, this method can be used to produce conjugates with good homogeneity [143,152].

#### 2.1.4. Glycoengineering Using Mutant Glycosyltransferases

Engineering of Fc glycosylation is a rational strategy to improve the safety and efficacy of therapeutic IgG antibodies. As mentioned above, glycans significantly influence the effector properties of the antibody, such as ADCC (antibody-dependent cellular cytotoxicity) and CDC (complement-dependent cellular cytotoxicity) [155,168]. Thus, by manipulating glycans, one can induce changes in these properties. In addition to the periodate oxidation of glycans and remodeling using natural enzymes, there are also various post-translational modification methods that employ mutant enzymes, like mutant β1,4-galactosyltransferase [169] and endoglycosidase Endo-S (endo-β-*N*-acetylglucosaminidase from *Streptococcus pyogenes*) [152]. These enzymes allow either the modification of the glycan structure followed by periodate oxidation [152], or the introduction of non-natural sugars bearing the target functional group into glycans. While there is a wide range of such groups available, this review will focus only on approaches related to the introduction of carbonyl groups [169].

One such approach is to introduce C2-keto-galactose into the antibody using a mutant β1,4-galactosyltransferase (Figure 10). This method allows for the introduction of a larger number of payload molecules, approximately 4, compared to 1.6 using the sialic acid residue introduction method described above [169].

An alternative method involves the chemoenzymatic synthesis of glycoengineered IgGs, including glycosite-specific antibody–drug conjugates (gsADCs), by almost complete reconstruction of the glycans [152,170,171,172]. This is achieved using the mutant Endo-S D233Q glycosynthase enzyme and pre-synthesized non-natural N-glycan oxazolines serving as substrates. Carbonyl groups can also be introduced via periodate oxidation for subsequent oxime ligation [170]. For a detailed account of N-glycan oxazoline synthesis, please refer to the review [173].

**Figure 11:**
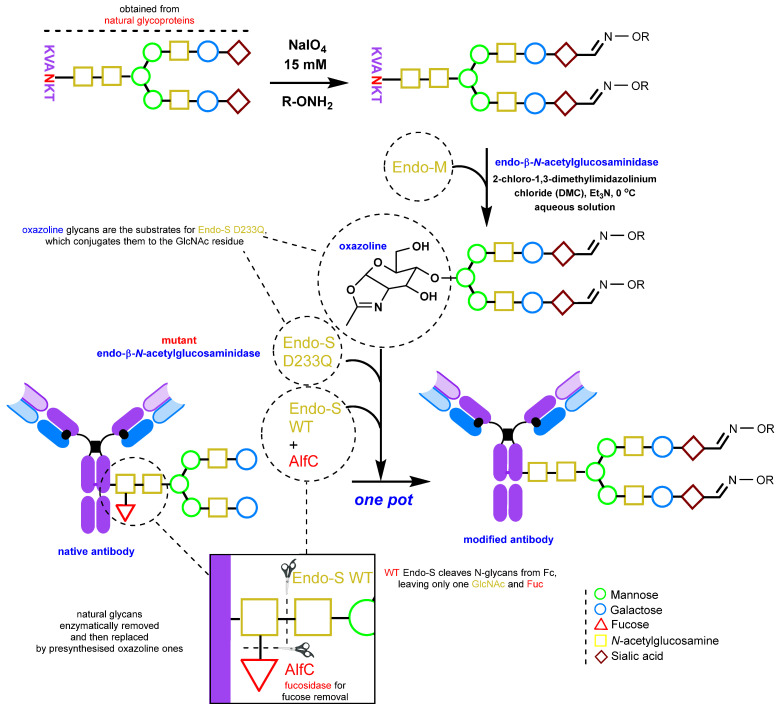
Enzymatic engineering of glycans [152,169,170,171,176].

This method uses the hydrolytic activity of the wild-type (WT) endoglycosidase Endo-S to cleave N-glycans from native immunoglobulins [174]. This step is crucial because, as mentioned above, natural IgGs have heterogeneous glycosylation and many glycan isoforms. After this enzymatic process, a single GlcNAc (*N*-acetylglucosamine) is left on the antibody (Figure 11) If defucosylation is required, fucosidase AlfC from *Lactobacillus casei* [175] can be added during the deglycosylation step. Next, another enzyme, mutant endoglycosidase (also known as glycosynthase), with high transglycosylation capacity but no hydrolytic activity, is used to attach the pre-synthesized N-glycan oxazoline bearing the requisite functional group (in the context of this review, the carbonyl group or its precursor) to the GlcNAc residue. Notably, this entire enzymatic procedure can be performed in one pot [170].

Endo-β-*N*-acetylglucosaminidase from *Streptococcus pyogenes* (Endo-S) is an endoglycosidase enzyme [174,175,177,178,179,180,181] that recognizes the N-glycans of IgG and has long been used for glycan remodeling of therapeutic antibodies. It accepts a wide range of glycans as substrates. Currently, a large number of mutant variants of the enzyme have been developed that allow mannose-rich and complex glycans to be attached to antibodies. Endo-S2 [171,172] or Endo-S D233Q [170] are two examples of such variants. The method of glycan engineering using mutant glucosaminidase has been the subject of numerous reviews and studies [115,182,183,184,185].

The mutant Endo-S D233Q uses oxazoline glycan derivatives as substrates (Figure 11). These derivatives can be obtained in a one-pot reaction from natural glycopeptides, such as egg yolk sialylglycopeptide (SGP), by using Endo m endoglycosidase from *Mucor hiemalis*. Following enzymatic glycan cleavage, oxazoline formation proceeds in an aqueous solution in the presence of 2-chloro-1,3-dimethylimidazolinium chloride (DMC) [170].

Incorporation of a carbonyl group into the free glycans can be accomplished by periodate oxidation using 15 mM NaIO_4_ in a phosphate buffer at pH 7. The subsequent oxime ligation must be performed before oxazoline synthesis [170]. Glycan oxidation and oxime ligation can be carried out in the same manner after glycan conjugation to the antibody, but with a lower concentration of periodate [176].

### 2.2. Introduction of Carbonyl Groups with Genetic Engineering Tools

#### 2.2.1. Introduction of a Natural Amino Acid as a Precursor of the Carbonyl Group

**Figure 12:**
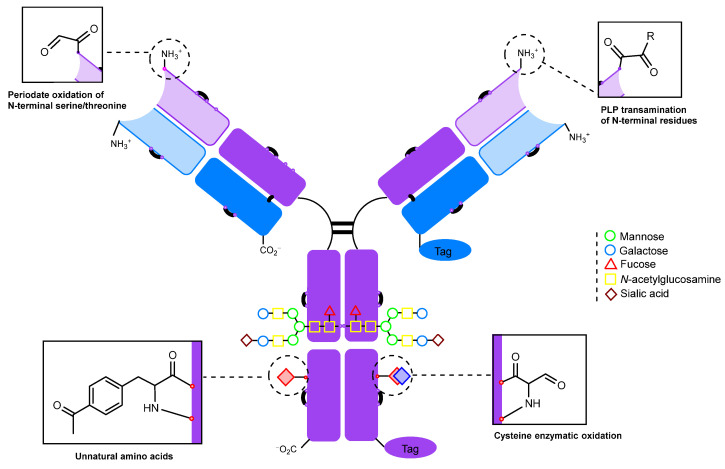
Potential modification sites of a genetically engineered antibody.

The production of recombinant monoclonal antibodies [186] is a well-established process [187,188] that has been extensively described in a number of reviews [188,189,190,191,192]. There are additional thorough reviews on bispecific antibodies [193,194,195], nanobodies [196,197], bi-, tri- and tetrabodies [198], and other derivatives [199,200]. This review will not cover these subjects. However, the production of recombinant monoclonal antibodies presents an opportunity to edit the amino acid sequence [201] and insert reactive natural amino acid residues into regions of interest [202,203] within the antibody to allow for further modification (Figure 12).

There are two main approaches to amino acid insertion:

- Inserting an amino acid residue of interest outside the complementarity-determining region (CDR) near the N-terminus [204].
- Placing an amino acid residue of interest into the Fc region or Fab region, far from the antigen-binding site [203]. Subsequent to this step, enzymatic or chemical conjugation can be performed.

Commonly inserted amino acids include serine, threonine, proline, selenocysteine [205], and cysteine. The introduction of cysteine residues, in particular, has gained popularity, often referred to under the trademark “THIOMAB” by Genentech [101]. This technology has been used in the development of FDA-approved antibody–drug conjugates such as “Polivy” (polatuzumab vedotin) [206] and the first-in-class antibacterial ADC for targeting *Staphilococcus aureus*, DSTA4637S [207], which is currently in clinical trials [208,209,210].

**Figure 13:**
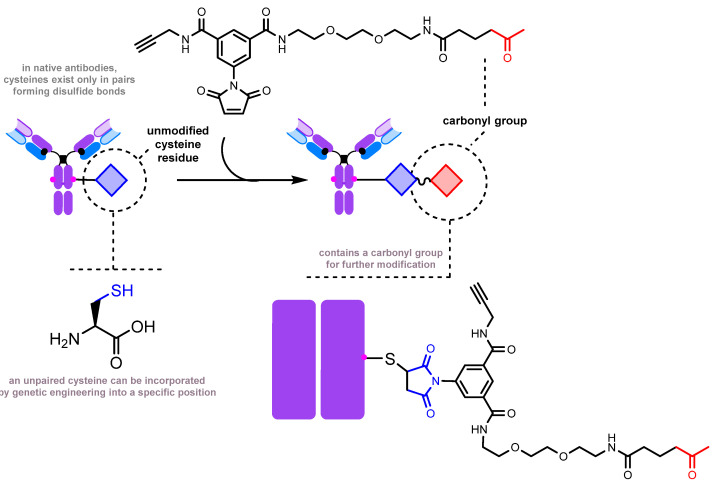
Ketone group introduction by thiol-maleimide chemistry [211].

The engineered THIOMAB antibody can be modified with a bifunctional maleimide-carbonyl-containing linker, which introduces a carbonyl group to the antibody for subsequent modification (Figure 13) [211]. In this case, the carbonyl group serves as one of the two reactive handles for the synthesis of a dual-drug antibody–drug conjugate [212].

**Figure 14:**
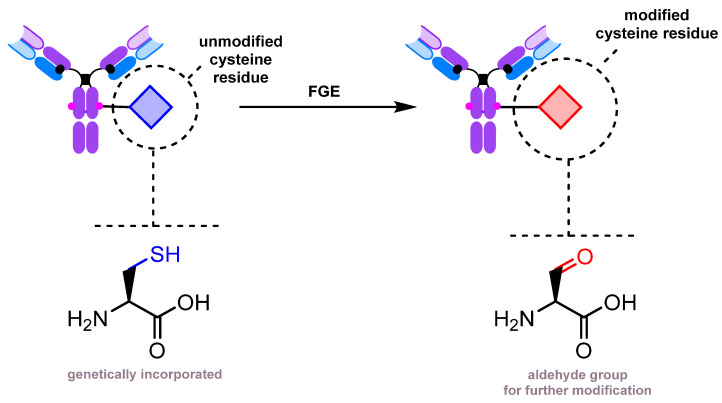
Enzymatic oxidation of cysteine to formylglycine [213,214].

Another strategy involves the enzymatic conversion of cysteine residues to formylglycine (Figure 14). It has been employed for modifying antibodies [213,214,215] and Fc fragments [216].

**Figure 15:**
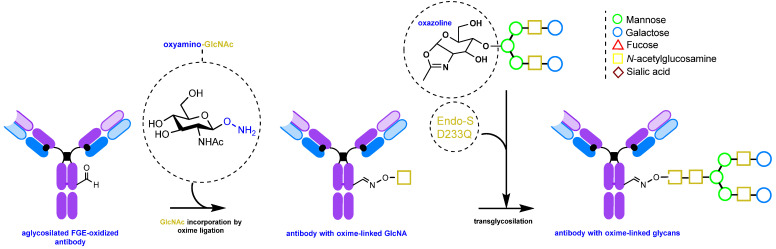
De novo glycan assembly on an aglycosylated antibody [217].

An interesting example of the vast possibilities of genetic engineering is an approach to glycan remodeling using the mutant Endo-S D233Q and a genetically engineered antibody containing a carbonyl group (Figure 15). The carbonyl group is introduced by enzymatic oxidation by first genetically encoding a cysteine residue in place of N297 and then converting it to formylglycine using FGE (formylglycine-generating enzyme), as described previously [217]. The formylglycine residue is further conjugated with an oxyamino GlcNAc derivative. The Endo-S-mediated conjugation with an N-glycan oxazoline completes the assembly process.

**Figure 16:**
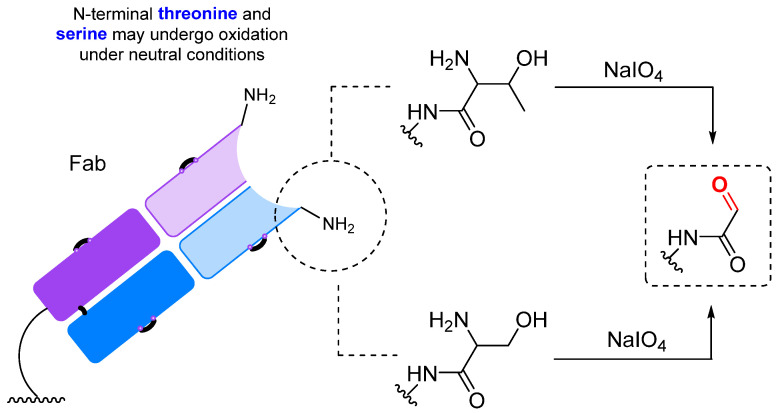
Periodate oxidation of N-terminal residues [204].

The N-terminal serine and threonine can be selectively oxidized with sodium periodate to form carbonyl compounds [204] (Figure 16). It should be noted that the rate of oxidation of α-amino alcohols and N-terminal amino acids with an OH group in the side chain is significantly higher (approximately 10^2^–10^4^ times) than the rate of oxidation of diols in sugars [218]. However, the rate of oxidation of amino alcohols is highly dependent on pH values. Under acidic conditions, the oxidation of amino sugars is almost completely suppressed due to the protonation of the amino group. In addition, as mentioned previously, at acidic pH valuesl, there is risk of methionine oxidation, while at neutral pH values, the oxidation of Met, Tyr, and Trp is virtually absent [136]. This makes it possible to selectively oxidize N-terminal residues under neutral conditions. In particular, periodate oxidation of the N-terminal serine of the IgG light chain followed by oxime ligation was used to synthesize a highly homogeneous antibody–drug conjugate with a DAR (drug-to-antibody ratio) of 2 [204].

#### 2.2.2. Introduction of a Protein Tag for Subsequent Carbonyl Group Insertion

The method of genetically engineered introduction of an amino acid sequence recognized by a specific enzyme—a tag—has found widespread application [219]. In particular, there are reports on the introduction of a carbonyl group into the antibody using an enzyme tag [220,221].

**Figure 17:**
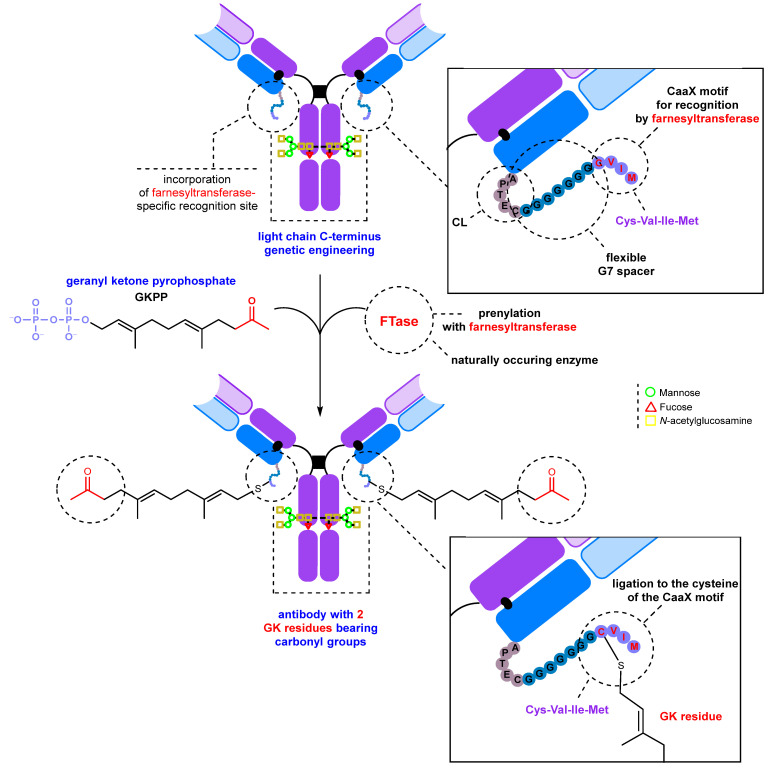
Introduction of a ketone group into the antibody using farnesyltransferase (FTase) [220,221].

The approach is based on the introduction of a flexible oligoglycine spacer (G7) and a specific CaaX (Cys-Val-Ile-Met) motif, which is recognized by farnesyltransferase (FTase, EC 2.5.1.58), into the C-terminus of the light chain of the antibody by genetic engineering. This motif is then used to attach geranyl ketone pyrophosphate to the antibody (Figure 17) [220,221]. This method is highly site-specific because prenylation occurs at the exact site recognized by the enzyme. It allows the introduction of two carbonyl-containing fragments into a single antibody molecule. The modification process does not adversely affect the properties of the antibody, and the modification site is located far from the antigen binding site, ensuring that there is no reduction in affinity. In addition, a different geranyl pyrophosphate analog bearing an aromatic aldehyde group was used to introduce a single modification into a DARPin (designed ankyrin repeat protein) for further conjugation with a fluorophore or a drug [222].

An unconventional approach for the site-specific introduction of an aldehyde group into nanobodies is based on the use of an oligohistidine tag [223]. The conjugation of a diol, which serves as a precursor for aldehyde generation, with a lysine residue is performed by reductive amination. The diol also carries a chelating moiety that, in the presence of Cu^2+^ ions, comes into close proximity with the histidine tag through complex formation, making the conjugation site-specific. In the next step, the diol is cleaved by 1 mM NaIO_4_, which simultaneously removes the reaction-directing metal-chelating moiety from the conjugate and generates an aldehyde group that can be used for conjugation to a dye or drug by either hydrazone formation or oxime ligation.

#### 2.2.3. UAAs Containing Carbonyl Groups

**Figure 18:**
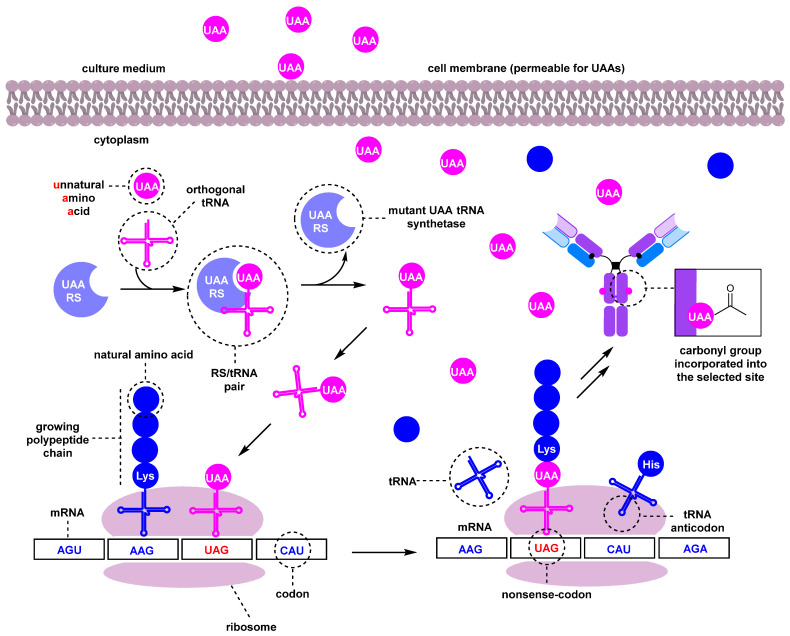
A simplified scheme of the cell-based UAA incorporation into antibodies [109,225,227].

A more complex method of introducing a carbonyl group into the antibody involves the use of unnatural amino acids (UAAs) [109]. These UAAs, which contain a carbonyl group, can be incorporated into the antibody at specific positions using genetic engineering [224]. There are two primary approaches to obtaining such genetically engineered antibodies: a cell-based method (Figure 18) and a cell-free method. A number of reviews are devoted to a detailed consideration of the characteristics of the methods [225,226]. In this review, we will only briefly discuss the cell-based method, as it is more commonly used and better developed in practice.

**Figure 19:**
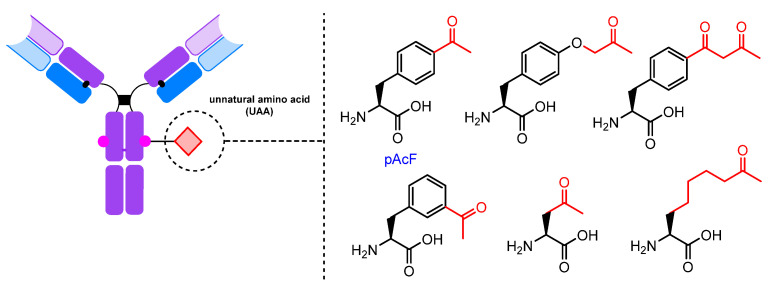
Carbonyl-containing UAAs.

The incorporation process begins with the recognition of an mRNA codon by a tRNA anticodon in the ribosome. The UAA is typically encoded by a nonsense codon in the mRNA, commonly UAG (amber); the codon choice depends on the host cells [228]. A specialized, orthogonal tRNA charged with the unnatural amino acid is capable of recognizing this codon and incorporating the UAA into the growing polypeptide chain. A mutant tRNA synthetase (RS) selectively acylates only the corresponding orthogonal tRNA with the unnatural amino acid. The selectivity of the tRNA/RS pair must be very high. Mutant RS/tRNA and antibody genes are introduced into *E. coli* cells with plasmids or into mammalian cells with vectors [225,227,229,230,231]. It is important that the amino acid containing the carbonyl group can cross the cell membrane and accumulate in the cytosol when added to the culture medium. Para-acetyl phenylalanine (pAcF) has been used to introduce the ketone group into antibodies [232], and a number of other carbonyl-bearing amino acids for incorporation into proteins have been reported [233,234,235,236,237] (Figure 19).

For example, a mutant anti-Her2 (human epidermal growth factor receptor 2) immunoglobulin containing pAcF was obtained in *E. coli* with the amber nonsense codon TAG using an orthogonal suppressor aminoacyl-tRNA synthetase/tRNA pair derived from *Methanococcus jannaschii* [228,232,238,239,240,241,242,243]. This approach has also been employed to generate bispecific antibodies targeting both HER2 and CD30 (TNFRSF8, tumor necrosis factor receptor superfamily member 8) [244], as well as Fab antibody fragments [240,242] and nanobodies [245] bearing a carbonyl group suitable for conjugation.

## 3. Conjugates: Modification of the Carbonyl Group

**Figure 20:**
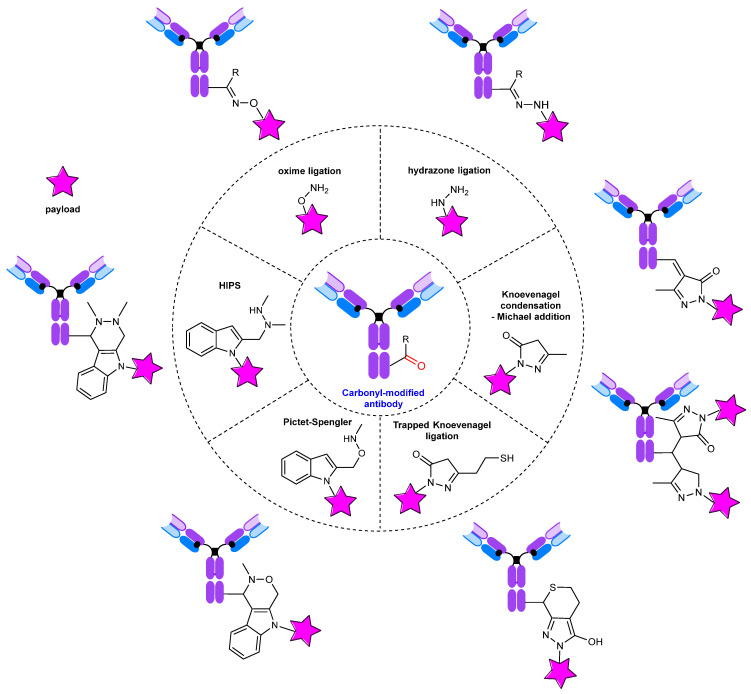
Modification of carbonyl-containing antibodies.

The presence of a carbonyl group in the antibody offers several possibilities for modification (Figure 20), including oxime and hydrazone formation [232], the Pictet–Spengler [246] and hydrazino–iso–Pictet–Spengler (HIPS) reactions [214], and the Knoevenagel condensation–Michael addition [247,248]. Several reviews are devoted to the modification of carbonyl-containing proteins and peptides [105,249,250,251].

Oxime ligation is considered to be an effective and reliable approach for protein modification [252]. In an extensive review published in 2017 [106], several aspects of the application of oxime ligation for bioconjugation, particularly those related to mechanism and catalysis, were thoroughly examined.

**Figure 21:**
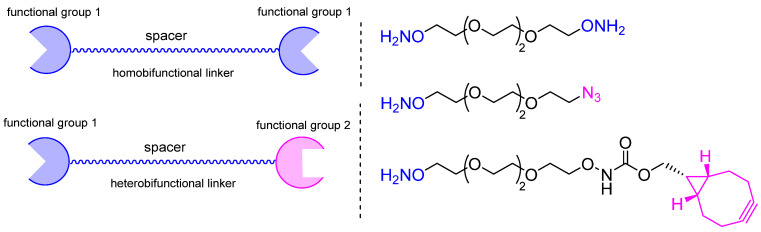
Examples of bifunctional linkers for oxime ligation.

The most straightforward technique for modifying immunoglobulins via oxime ligation involves conjugation using a long hydrophilic oxyamine linker (Figure 21). This linker can carry functional groups, such as azide [170,244,253,254], cyclooctyne [170,254], alkyne [170], or oxyamine [231], for further biorthogonal modification.

**Figure 22:**
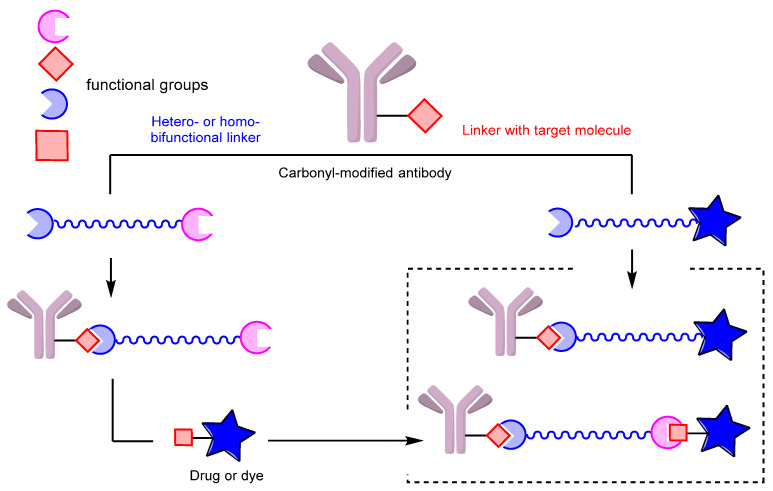
Two approaches to linker-based conjugation of carbonyl-modified antibodies; **left**: sequential assembly, **right**: one-step ligation.

An alternative approach involves directly conjugating the target molecule with the antibody in one step, eliminating the need for additional procedures (Figure 22).

**Figure 23:**
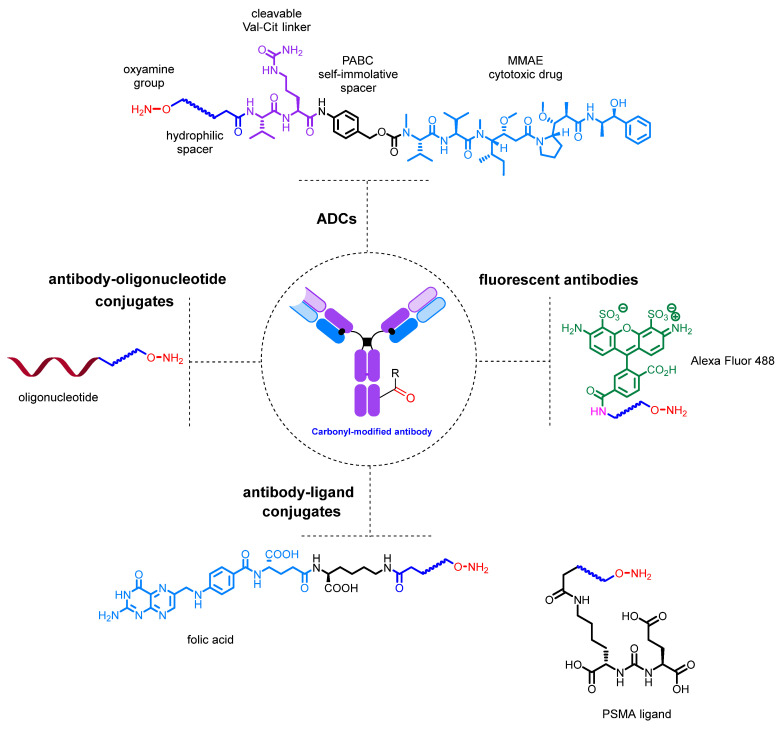
Examples of conjugates obtained by single-step conjugation.

Antibody conjugates with dyes [137,216,232,254,255], cytotoxic drugs such as auristatins [169,221,241,243,256,257] and pyrrolobenzodiazepines [220], folic acid [258], PSMA (prostate-specific membrane antigen) ligands [242], and LXR (liver X receptor) agonists [259] have been generated using this approach. In addition, numerous antibody conjugates with oligonucleotides have been developed, demonstrating the versatility of this method [238,239,240,260] (Figure 23).

## 4. Conclusions

**Figure 24:**
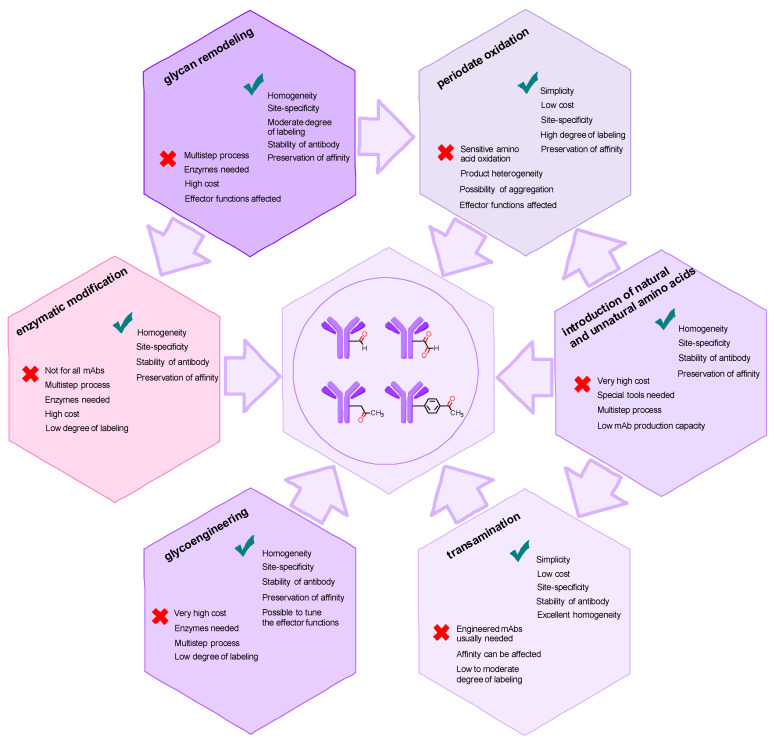
Methods for introducing carbonyl groups into antibodies and the relationships between them.

The introduction of a carbonyl group into an antibody is an established method for the production of antibody conjugates. There are a wide variety of approaches, ranging from simple chemical oxidation to the incorporation of noncanonical amino acids requiring specialized expression platforms (Figure 24).

Periodate oxidation is the oldest and, arguably, the most versatile method for generating reactive aldehyde moieties from either glycans, synthetic diols or N-terminal amino acids. Although many side reactions, including antibody oligomerization and undesired amino acid oxidation, are possible, they can be limited by careful control of reaction conditions. As a result, NaIO_4_ remains a suitable reagent choice, especially when combined with modern antibody modification methods for milder oxidation conditions and product homogeneity. Another chemical oxidation method, transamination mediated by PLP and related reagents, despite some drawbacks, represents a convenient strategy provided that the N-terminal residues can be cleanly converted to α-oxo aldehyde moieties. That being said, the N-terminal regions of any antibody can be engineered using standard procedures, so residues suitable for transamination can be introduced, rendering the approach general, albeit, not perfect, as complete homogeneity of the conjugate is hard to achieve due to side reactions.

Enzymatic modification of N-glycans in the Fc fragment can range from simple manipulation of terminal sugar residues using conventional enzymes to radical rebuilding of the entire glycan core using specially designed mutant enzymes with the purpose of introducing new functionality. The complexity of the methods increases with the depth of the oligosaccharide modification, but, in all cases, the exceptional chemoselectivity of the enzymatic reactions allows for the generation of highly homogeneous products. Complete reengineering of the Fc glycan has the added benefit of tailoring antibody effector properties. This can play a role in enhancing the efficacy of glycosite-specific antibody–drug conjugates with ADCC and CDC.

Genetic engineering offers vast possibilities for antibody modification in general and the introduction of reactive carbonyl groups in particular. The more conventional approaches involve the introduction of unpaired cysteines (the THIOMAB technology), which are further converted to formylglycine residues, and the addition of a protein tag for enzymatic or affinity-guided conjugation. A much more laborious but elegant strategy is based on the introduction of unnatural amino acids containing a keto group. Upon further modification of the carbonyl group, all of these methods give rise to site-specific, homogeneous antibody conjugates.

It is important to note that there are links between many of these methods. For instance, enzymatic glycan modification is often followed by periodate oxidation, while glycan remodeling itself may rely on antibody sequence engineering. In another example, engineering the N-terminus can assist in transamination or allow selective periodate oxidation when serine or threonine are introduced.

In recent years, numerous approaches to the introduction of reactive carbonyl groups into antibodies and their derivatives have been proposed. We expect this trend to continue, as carbonyl-based conjugations, particularly hydrazone formation and especially oxime ligation, represent an important tool in the toolbox of biorthogonal reactions. Rivaling the extremely well-developed azide–alkyne cycloadditions in terms of selectivity and reaction rate, oxime ligation is continuously being used to generate antibody conjugates, in particular, ADCs.

In a global sense, antibody–drug conjugate technologies are currently focused on obtaining conjugates that are as site-specific and homogeneous as possible. Equally important is the stability of the conjugate in the bloodstream, which includes the reliability of conjugation of the linker with the cytotoxic agent to the antibody as well as the stability of the linker in the plasma. The homogeneity and site-specificity of the ADC, as well as its stability in plasma, significantly improve its pharmacodynamic properties, efficacy, and reduce the severity of side effects.

It appears that the most effective approach involves incorporating a bioorthogonal functional group into antibodies through the use of natural and genetically engineered amino acids. This approach is well established for antibody conjugates produced by the THIOMAB technology, which consists of introducing an additional unpaired cysteine into the antibody that is suitable for further modification, including the generation of a carbonyl group by FGE. One of the conjugates produced using this technology, Polivy, has already been approved by the FDA for use in patients. Several others, including an antibacterial conjugate, are in clinical trials.

The introduction of unnatural amino acids with a carbonyl group, such as para-acetylphenylalanine, is also a promising approach. However, unlike the introduction of natural amino acids into the antibody sequence, the introduction of unnatural amino acids with biorthogonal functional groups is a very labor-intensive task: it requires obtaining a mutant tRNA/RS pair, which is not trivial in itself, and overcoming difficulties with antibody production in cell culture. This makes the process expensive and makes it difficult to apply the method to the production of conjugates on an industrial scale. Nevertheless, a total of three ADCs currently in clinical trials (ARX788 [261], ARX517 [262], and AGS62P1 [256] are produced by this method with pAcF introduction followed by oxime ligation with an oxyamine-containing auristatin-based payload.

We believe that genetically engineered antibodies will come to the fore in the future as the technology of their production develops. The advantages of the method, in addition to those mentioned above, include the convenience of the conjugation procedure, reproducibility, and the ability to introduce the amino acid residue strictly at the required position on the antibody, since the conjugation site also has a significant influence on the properties of the final conjugate. The use of genetically engineered amino acids is probably the only conjugation method currently available that allows one to choose the site on the antibody where the cytotoxic drug molecule will be introduced.

Another interesting method worth mentioning is glycan remodeling, also known as GlycoConnect. The advantages of this method include the ability to edit glycans of any off-the-shelf antibody. Glycans are known to play an important role in the interaction of antibodies with the immune system, as well as influencing the circulation time of the antibody in the bloodstream. The ability to model antibody glycans and thus modify the properties of the conjugate seems promising. In addition, the method is site-specific since the modification of the antibody is performed strictly on glycans and allows to obtain homogeneous conjugates with a strictly defined loading. Several such conjugates, such as ADCT-601 [263], are currently in clinical trials.

The antibody–drug conjugates currently used in patients are mostly older-generation drugs that were introduced into clinical trials many years ago. In recent years, ADCs based on outdated technologies, particularly statistical labeling of lysines and cysteines, have also entered the market. We expect that the ADC market will continue to be supplied with technologically obsolete examples of therapeutic antibody conjugates for some time to come, as it takes at least ten years from conjugate development to clinical testing and approval. In the future, we expect a number of new site-specific and homogeneous conjugates to enter the market with improved properties and less severe side effects, including those produced by carbonyl-based conjugation.

## Data Availability

Not applicable.
